# Improving recruitment into psychiatry: teaching strategies to enhance undergraduate interest

**DOI:** 10.3402/meo.v17i0.19223

**Published:** 2012-11-30

**Authors:** Vic Marimuttu, Nisha Chandwani

Like many medical disciplines, interest in the practice of psychiatry is subject to a myriad of social and economic forces. Perhaps more so than most specialties, current events – and the manner in which they are reported – undoubtedly influence how mental illness and, subsequently, psychiatry are publicly viewed. The recent tragic shooting incident at a cinema in the United States, and the ‘disclosure’ that the shooter had seen a psychiatrist, exemplifies the media's often sensational interest in mental illness. Indeed, mental health is brought to the fore by the media time and time again in coverage that often overgeneralizes mental illness and, on occasion, may not be fully accurate. Such portrayals may not only further stigmatize sufferers of mental illness but also those who work or are contemplating careers in the field of psychiatry.

The effects of the anti-psychiatry movement in the 1960s have been far reaching, affecting the views of many that several mental illnesses are social constructs with little, if any, biological underpinnings. Although neurobiological research has since made compelling scientific challenges to this standpoint, the legacies of Szasz, Laing, and Foucault (for example), as well as other influential thinkers, remain poignant. Subsequently, it is perhaps not surprising that psychiatry is often viewed as a comparatively non-scientific and less efficacious career choice amongst medical graduates.

Recruitment into psychiatry is a global challenge. In their national survey of United Kingdom (UK) graduates and their career choices in the period 1974–2000, Goldacre and associates (1) found that only 4–5% of graduates chose psychiatry – a percentage that has remained largely unchanged over the years. In the United States, recruitment has been gradually decreasing since the 1970s, reaching its lowest in 1994, with just 3.2% of medical graduates choosing psychiatry (2). In a more recent survey of second year Australian medical students (3), only 15% of respondents indicated a strong likelihood of choosing psychiatry as a specialty. In contrast, other specialties attracted higher proportions of students in the range of 19–49% (3). One of the main reasons for choosing psychiatry was learners’ experience of the subject as medical students – substantiating the claim that ‘the psychiatry clerkship is the most important medical school influence on recruitment’ (2).

## Is teaching of psychiatry good enough?

Weintraub and colleagues believed that an ‘enthusiastic psychiatric faculty intimately involved with students over an extended period of time was the crucial factor in neutralizing anti-psychiatric socialization experiences in medical school’ (4). This ‘attachment’ can be a useful opportunity to engage in discussions around the positive and negative views of mental illness and psychiatry by the wider medical faculty, the media, and society in general. Unfortunately, many memories of psychiatry clerkship training involve dull ward rounds, sitting in on outpatient clinics, and attending mundane didactic lectures.

In psychiatry, there is clearly no equivalent to palpating for hepatomegaly. However, like the subtle nuances of abdominal palpation, it is not the quantity of exposure that renders one's training memorable, but rather the quality of the context within which it is initially experienced. Toward this end, nothing can completely replicate the experience of seeing and examining patients under the guidance and supervision of a practicing clinician. Like other medical disciplines, direct supervision and observation accompanied by teaching remains the preferred model of clinical instruction in psychiatry, although such high-quality teaching is time consuming and often conflicts with service provision commitments. However, this learning experience – and the structural barriers to readily instilling it – are hardly unique to psychiatry. Why then, does the field seem to differentially bear the burden of these effects?

## How can we improve teaching to engage students and increase recruitment?

Clearly, an attitudinal shift is required to improve the way we teach. We need to embrace the challenges of teaching psychiatry and find innovative ways to help students learn while simultaneously capturing their interest and stimulating their curiosity. One particular pedagogy that holds promise is that of role-play, where students assume the roles of patients and providers. What follows are some ideas for how such approaches may be used to improve teaching in psychiatry.

### Role-play

Role-play can involve trainee psychiatrists as actors, as they have a wealth of experience and are able to act out various illnesses, lending realism to what is often a fairly artificial interaction. Such sessions could go a step further by being done in a consulting room, with closed circuit television feeding video into a nearby/next door room, where other students are seated. It is important that feedback following review of the video is provided, and that each student has an opportunity to take part in the role-play.

Such exercises are not sufficient to cover the entire curriculum. However, if done well, they can serve as drivers for students to take a greater interest in psychiatry, and allow them the space to discover an aptitude for such work. One such example is by having the student interview an actor who is having suicidal thoughts, and has an underlying depressive illness, only to repeat the role-play with the same actor and same script, but with the actor playing the persona of a person with borderline personality disorder. Although similar symptoms are elicited in both role-plays, the exercise not only helps in the teaching of the mental state examination but also provides a discussion for the various feelings and emotions experienced by the interviewer when interviewing a person who is depressed, and one who is having a personality disorder. Our experience of using such contrasting role-plays with students suggest that it was a useful exercise that highlighted the idea that psychiatry was not about eliciting a checklist of symptoms but rather more about paying close attention to the mental state examination. Students also found it a useful way of understanding the concepts of transference and countertransference.

### Media

As previously alluded to, much material in psychiatry is fundamentally different from that in medicine or pediatrics (for example), making it crucially important to ‘contextualize’ key concepts in a readily understood manner. Textbooks can only give a limited picture of any disease or disorder. However, if properly done, the integration of popular media can help ‘flesh out’ the dimensionality of such concepts, thus improving and consolidating learning.

For example, in the popular American situation comedy titled ‘The Big Bang Theory’, one of the main characters is commonly assumed to have an autistic spectrum disorder. This is a disorder where the various traits can be difficult to grasp if simply recited in a textbook. However, when viewed in context of popular (albeit fictional) culture, constituent concepts can be more easily identified, linked, and explained – making the learning process more engaging and, perhaps, increasing retention of key concepts. Using popular media for clinical education purposes also allows the instructor to be creative and playful while encouraging discussion. Further, using popular fictional families such as ‘The Simpsons’ or ‘The Sopranos’ allows for discussions around the role of family dynamics, parenting styles, sibling relationships, alcoholism, drug (ab)use, and other broader social issues that play an important role in precipitating and maintaining mental illnesses.

One such example involves an episode of *‘The Simpsons’* where Bart is diagnosed (by a non-clinician) with attention deficit disorder and is prescribed an experimental drug for attention deficit hyperacitivity disorder (ADHD) called Focusyn (5) – which causes him to become psychotic. The clip not only highlights concerns regarding the medicalization of oppositional behavior and the negative effects of over-medication but also shows the benefits of stimulants on ADHD symptoms. More importantly, despite some medical inaccuracies, the clip generates discussion of some important areas of psychiatric diagnosis and treatment. Of course, care must be taken in the choice of media examples, as there is a risk of further stigmatizing psychiatry with the use of inaccurate representations of mental illness.

### Technology

Currently, several universities in the UK use Websites (virtual learning environments) to post lecture slides, timetables, and discussion forums (for example) – requiring students to actively (personally assume the initiative) access materials via their computers. However, current trends largely pertain to ‘push’ technology, where information is routinely relayed to users’ mobile devices, instead of them having to access it. The rationale is that one is more likely to use information that is readily available, versus having to go looking for it.

Our suggestion is that students can subscribe to audio and video podcasts of lectures, using platforms such as iTunes U or YouTube. Another suggestion is that universities develop mobile apps that ‘push’ the latest lectures, videos, and learning objectives to learners’ mobile devices – allowing ‘whenever, wherever’ access on mobile devices such as smartphones and tablets. An undergraduate program that has an accessible and comprehensive virtual learning environment that ‘pushes’ the latest lectures/videos in a given area (like psychiatry) is likely to generate increased interest in that subject.

### Extended exposure

The nature of mental illnesses is typically chronic and longstanding, with repeated courses of relapse and remittance (e.g., depression, bipolar affective disorder, or schizophrenia). The nature of such disorders typically require longer periods of engagement to observe and recognize a patient's ‘full journey’. During short educational engagements, getting more intimately involved in a patient's illness experience may be difficult. As a result, students may get only a brief ‘cross-sectional’ snapshot of a patient's journey through a mental illness.

One possible remedy is to match first-year medical students to a patient with a chronic mental illness (e.g., depression) – with the intent that students periodically meet with and follow the patient longitudinally across their undergraduate medical training. Engaging students with patients’ ‘real-life’ stories and experiences may offer an effective means of improving their understanding of mental illness and, ultimately, nurturing their interest in psychiatry.

## Conclusion

In the UK and other parts of the world, recruitment into psychiatry has lagged behind other specialties for some time. It has been shown that undergraduate teaching is key to recruitment. In keeping with the times, medical education needs to be interesting, engaging, and effective for students. We believe that there is room for the use of role-play, media, and technology to improve the experience of medical students learning about the practice of psychiatry. Similarly, offering an extended psychiatric attachment can further help to make the experience practical and memorable for students, eventually leading to improved recruitment.

None of this is likely to occur without some investment of human, technological, and financial resources. Also, while role-play with medical students has been used to simulate doctor–patient interactions, it is not certain if such role-play (for example), even when scripted, can comparably emulate more realistic, less contrived scenarios. Role-play with actors, on the contrary, can be expensive and difficult to source. Our anecdotal feedback with such teaching has been largely positive. However, we believe more can be done to broaden the boundaries of ‘traditional’ approaches to combat some of the barriers keeping students from realizing a professional interest in psychiatry.
